# Biventricular Longitudinal Strain Predict Mortality in COVID-19 Patients

**DOI:** 10.3389/fcvm.2020.632434

**Published:** 2021-01-18

**Authors:** Yuji Xie, Lufang Wang, Meng Li, He Li, Shuangshuang Zhu, Bin Wang, Lin He, Danqing Zhang, Yongxing Zhang, Hongliang Yuan, Chun Wu, Wei Sun, Yanting Zhang, Li Cui, Yu Cai, Jing Wang, Yali Yang, Qing Lv, Mingxing Xie, Yuman Li, Li Zhang

**Affiliations:** ^1^Department of Ultrasound, Tongji Medical College, Union Hospital, Huazhong University of Science and Technology, Wuhan, China; ^2^Hubei Province Key Laboratory of Molecular Imaging, Wuhan, China

**Keywords:** COVID-19, speckle tracking echocardiography, strain, left ventricular function, right ventricular function

## Abstract

**Background:** Biventricular longitudinal strain has been recently demonstrated to be predictive of poor outcomes in various cardiovascular settings. Therefore, this study sought to investigate the prognostic implications of biventricular longitudinal strain in patients with coronavirus disease 2019 (COVID-19).

**Methods:** We enrolled 132 consecutive patients with COVID-19. Left ventricular global longitudinal strain from the apical four-chamber views (LV GLS_4CH_) and right ventricular free wall longitudinal strain (RV FWLS) were obtained using two-dimensional speckle-tracking echocardiography.

**Results:** Compared with patients without cardiac injury, those with cardiac injury had higher levels of coagulopathy and inflammatory biomarkers, higher incidence of complications, more mechanical ventilation therapy, and higher mortality. Patients with cardiac injury displayed decreased LV GLS_4CH_ and RV FWLS, elevated pulmonary artery systolic pressure, and higher proportion of pericardial effusion. Higher biomarkers levels of inflammation and cardiac injury, and the presence of pericardial effusion were correlated with decreases in LV GLS_4CH_ and RV FWLS. During hospitalization, 19 patients died. Compared with survivors, LV GLS_4CH_ and RV FWLS were impaired in non-survivors. At a 3-month follow-up after discharge, significant improvements were observed in LV GLS_4CH_ and RV FWLS. Multivariate Cox analysis revealed that LV GLS_4CH_ [hazard ratio: 1.41; 95% confidence interval [CI]: 1.08 to 1.84; *P* = 0.011] and RV FWLS (HR: 1.29; 95% CI: 1.09–1.52; *P* = 0.003) were independent predictors of higher mortality in patients with COVID-19.

**Conclusions:** LV GLS_4CH_ and RV FWLS are independent and strong predictors of higher mortality in COVID-19 patients and can track improvement during the convalescent phase of their illness. Therefore, biventricular longitudinal strain may be crucial for risk stratification and serial follow-up in patients with COVID-19.

## Introduction

Coronavirus disease 2019 (COVID-19), which is caused by the severe acute respiratory syndrome coronavirus 2 (SARS-CoV-2), has become a pandemic health crisis. Although, there is increasing awareness of the cardiovascular involvement in COVID-19 disease and its adverse impact on prognosis (1, 2), there is limited data regarding cardiac abnormalities due to SARS-CoV- 2 infection. Echocardiography remains the mainstay imaging modality for assessing cardiac function in clinical practice. Recently, left ventricular (LV) and right ventricular (RV) longitudinal strain measured by two-dimensional speckle-tracking echocardiography (2D-STE) has been proposed as more accurate and sensitive indicators of cardiac function in a variety of cardiovascular diseases (3–5). Furthermore, a number of studies confirmed the prognostic value of biventricular longitudinal strain in various clinical settings (6–8). However, the prognostic implications of biventricular longitudinal strain in COVID-19 patients has not been well-established. Accordingly, our study aimed to investigate whether biventricular longitudinal strain were independently predictive of higher mortality in patients with COVID-19 and explore their utility in the follow-up in these patients.

## Methods

### Study Population

This single-center, prospective study was performed at the west branch of Union Hospital, Huazhong University of Science and Technology, China, which was a designated hospital to treat patients with COVID-19. We enrolled 169 consecutive adult patients who were diagnosed with COVID-19 according to interim guidance of World Health Organization, from February 11 to March 16, 2020. Considering the presence of cardiac involvement in COVID-19 patients, bedside echocardiography was performed in all patients from three wards managed by the investigators for evaluation of cardiac function. The median time from admission to echocardiographic assessment was 7 days [interquartile range [IQR] 3–11]. Among these patients, three had dilated cardiomyopathy, four had old myocardial infarction, and 30 did not have images of sufficient quality for STE analysis. Finally, 132 patients were recruited in our analysis.

This study was approved by Union Hospital, Tongji Medical College, Huazhong University of Science and Technology Ethics Committee (KY-2020-02.06). Written informed consent was waived for all participants with emerging infectious diseases. Patients or the public were not involved in the design, conduct, reporting or dissemination plans of our research.

### Data Collection

Demographic characteristics, comorbidities, laboratory findings, medical history, complications, and outcomes for patients during hospitalization were independently reviewed by a team of trained physicians from electronic medical records. The timing of laboratory measurements were within 3 days of echocardiographic examinations with a mean interval of 1 days (IQR: 1–2). Acute cardiac injury was defined as serum levels of cardiac high-sensitivity troponin I (hs-TNI) above the 99th-percentile upper reference limit. The outcome was defined as in-hospital death. The final date of follow-up outcome were April 9, 2020.

### Transthoracic Echocardiography

Bedside transthoracic echocardiographic examinations were performed using an EPIQ7C machine (Philips Medical Systems, Andover, MA, USA) at the designated COVID-19 isolation wards or intensive care units (ICU). Forty-six survivors underwent follow-up echocardiographic examinations at 3 months after discharge. All scans were conducted by trained individuals in full personal protective equipment. All echocardiographic images were stored in digital format and analyzed by two independent observers (C.M. and Y.Z.) who were blinded to epidemiological and clinical characteristics, laboratory findings, treatment, and outcomes.

### Conventional Echocardiographic Analysis

Left ventricular (LV) and right ventricular (RV) structural and functional parameters were measured based on the guidelines of the American Society of Echocardiography (9). LV mass was assessed by the Devereux's formula. LV volumes and ejection fraction (EF) were obtained using Simpson's biplane method. LV diastolic function was assessed by the ratio of peak early-diastolic transmitral inflow velocity (E) to late-diastolic inflow velocity (A), and the ratio of transmitral E to the peak early-diastolic mitral annual velocity (e′). We also measured the deceleration time (DT) of the E-wave.

Tricuspid annular plane systolic excursion (TAPSE) was measured on M-mode echocardiography. RV fractional area change (RVFAC) was calculate as (RV end-diastolic area -RV end-systolic area)/end-diastolic area × 100%. Tricuspid lateral annular systolic velocity (S′) was assessed by tissue Doppler imaging from the apical 4-chamber view. Pulmonary artery systolic pressure (PASP) was evaluated using the simplified Bernoulli equation and right atrial pressure assessed on the basis of the size and collapsibility of the inferior vena cava.

### STE Analysis

**Figure 1:**
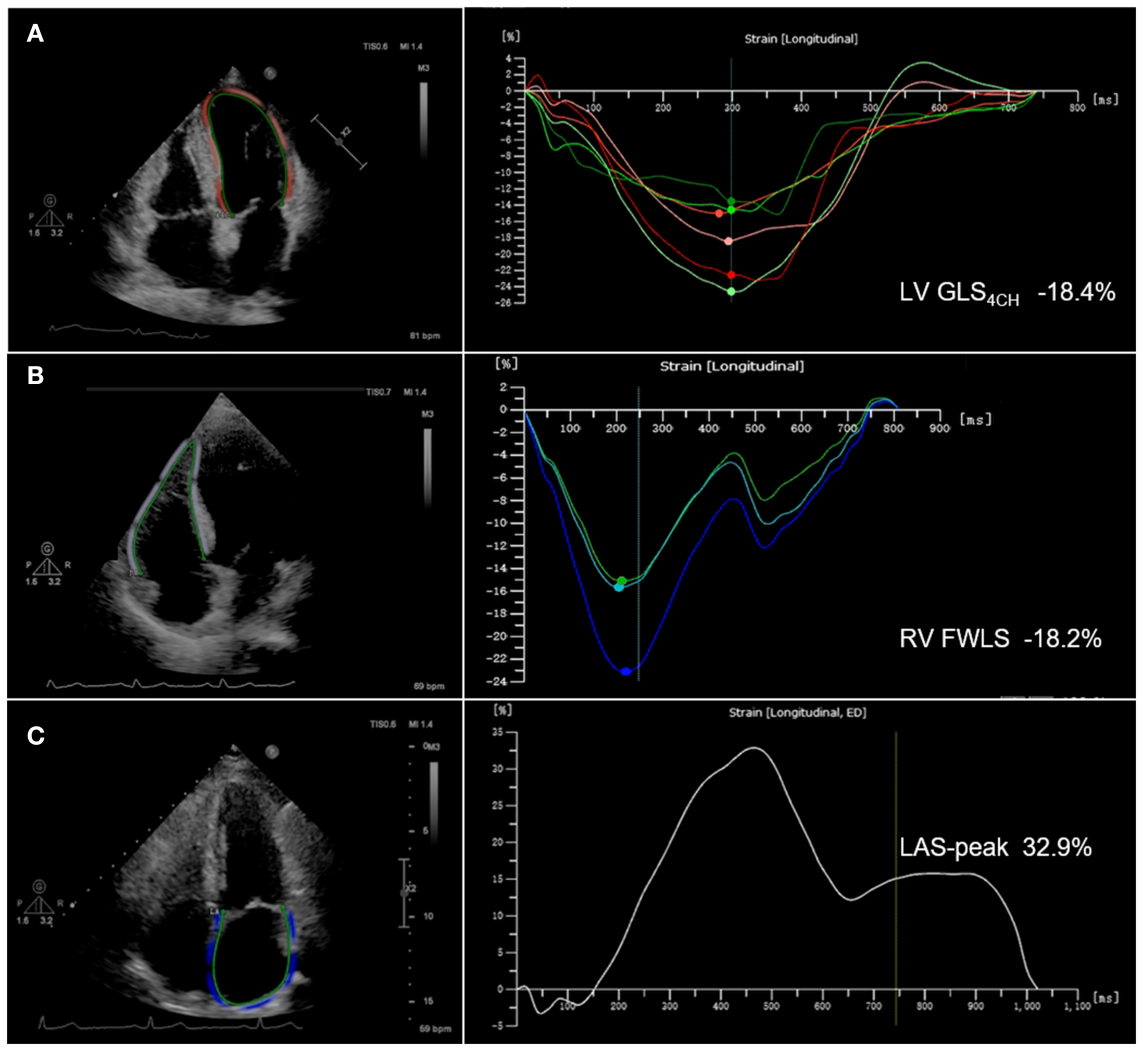
Biventricular and left atrial longitudinal strain obtained from two-dimensional speckle-tracking echocardiography in COVID-19 patients. **(A)** Representative image with left ventricular global longitudinal strain from the apical 4-chamber view (LV GLS_4CH_); **(B)** Representative image with right ventricular free wall longitudinal strain (RV FWLS); **(C)** Representative image with peak left atrial strain (LAS-peak).

STE analyses were performed using commercially available AutoStrain software (Qlab13, Philips Healthcare, Andover, MA, USA). LV global longitudinal strain (GLS) was calculated by averaging the values obtained in the apical 4-chamber, 3-chamber and 2-chamber views. LV GLS_4CH_ was defined as the mean of the strain values in the six segments of left ventricle from the apical 4-chamber view. LV GLS and GLS_4CH_ were obtained from the standard two-dimensional gray-scale image with a frame rate of 50~70 frames/s. The LV endocardial border was automatically traced at end diastole. Subsequently, the software tracked the endocardial layer throughout the cardiac cycle. The operator could manually adjust the endocardial border if necessary. Right ventricular free wall longitudinal strain (RV FWLS) was obtained from the standard two-dimensional gray-scale image of the RV-focused apical four-chamber view with a frame rate of 50~70 frames/s. The RV endocardial border was automatically traced at end diastole. The software tracked automatically the endocardial layer throughout the cardiac cycle and the observer may manually adjust the endocardial border if necessary. RV FWLS was calculated as the average of the basal, mid and apical RV free wall segments. Left atrial (LA) endocardial contours were drawn in the apical 4-chamber view with a frame rate of 50~70 frames/s at end systole. The appendage and pulmonary veins were not included. The endocardial border was automatically tracked by software throughout the cardiac cycle. Manual adjustments were performed when tracking was suboptimal. Peak left atrial strain (LAS-peak) was automatically generated from the software. Patients with two or more inadequately tracked segments were removed from analysis. Representative images with LV GLS_4CH_, RV FWLS and LAS-peak are shown in Figure 1. Absolute values of LV GLS, GLS_4CH_ and RV FWLS were presented in this study for a simpler interpretation, as LV GLS, GLS_4CH_ and RV FWLS were negative values.

### Interobserver and Intraobserver Reproducibility

Intraobserver and interobserver variability of LV GLS_4CH_, RV FWLS and LAS-peak were estimated in 20 randomly selected subjects and evaluated by intra-class correlation coefficient (ICC) and Bland-Altman analysis. Intraobserver variability was evaluated by having one observer remeasure after 4 weeks. Interobserver variability was assessed by a second observer who was blinded to the first observer's measurements.

### Statistical Analysis

Continuous numeric variables were expressed as mean ± SD or medians (IQR) and compared using a two-sample Student's *t*-test and one-way analysis of variance for normally distributed data, or Mann-Whitney test and Kruskal-Wallis test for non-normally distributed data. Categorical variables were expressed as frequency (percentage), and compared using the χ^2^ test or Fisher's exact test. Spearman's correlation coefficient were used to evaluate the association between biventricular strain and laboratory findings. Univariate and multivariate Cox regression models were used to assess the predictors of higher mortality. All potential predictors of higher mortality were included into univariate analyses: age, gender, comorbidities, complications, laboratory findings and echocardiographic parameters. Variables with *P* < 0.05 at univariate analysis were entered into multivariate Cox regression models. Owing to smaller patients with endpoints, there may exist an over-fitting issue. Therefore, to avoid problems of overfitting the data, a separate Cox proportional hazard model including clinical variables and each of biventricular function parameters (LV GLS_4CH_, TAPSE, RVFAC, and RV FWLS), was used to determine the independent predictors of higher mortality. The model performance was assessed by Akaike Information Criterion (AIC). Receiver operator characteristic (ROC) curves were used to determine the optimal cutoff value of LA, LV and RV function parameters for detecting poor outcomes. Kaplan-Meier survival curves were plotted and compared using the log-rank test. All statistical analyses were performed using a SPSS version 20.0 (SPSS Inc., Chicago, Illinois), and a two-sided value of *P* < 0.05 was considered as statistically significant.

## Results

### Clinical Characteristics

Clinical characteristics of patients with COVID-19 are presented in Table 1. The mean age of patients was 61 ± 13 years, and 68 (51.5%) were male. Of the 132 patients, 40 (30.3%) patients displayed acute cardiac injury. Compared with patients without cardiac injury, those with cardiac injury had lower lymphocyte count, and higher levels of coagulopathy and inflammatory biomarkers [prothrombin time (PT), activated partial thromboplastin time (APTT), C-reactive protein (CRP), procalcitonin (PCT) and interleukin 6 (IL-6)]. The levels of creatine kinase muscle-brain (CK-MB) and B-type natriuretic peptide levels were also higher in patients with cardiac injury than those without. Additionally, patients with cardiac injury were more likely to develop acute kidney injury and acute respiratory distress syndrome (ARDS), and be admitted to ICU. And they were more likely to receive treatment with high-flow oxygen and mechanical ventilation, and had higher mortality.

**Table 1:** Baseline clinical characteristics of patients with COVID-19 according to acute cardiac injury.

**Variables**	**All patients (*n =* 132)**	**Without cardiac injury (*n =* 92)**	**With cardiac injury (*n =* 40)**	***P*-value**
**Clinical characteristics**				
Age, years	61 ± 13	60 ± 13	63 ± 12	0.176
Male, *n* (%)	68 (51.5%)	43 (46.7%)	25 (62.5%)	0.096
Body mass index, kg/m^2^	23.6 ± 2.9	23.6 ± 2.9	23.8 ± 3.0	0.653
Heart rate, beats/min	86 (80,102)	86 (80,100)	90 (80,107)	0.143
Respiratory rate, breaths/min	23 (20,30)	23 (20,30)	24 (20,29)	0.966
SBP, mm Hg	132 (120,144)	132 (121,144)	131 (115,146)	0.735
DBP, mm Hg	80 (73,87)	80 (75,89)	80 (72,85)	0.235
Smoker, *n* (%)	6 (4.5%)	4 (4.3%)	2 (5.0%)	0.591
**Comorbidities**				
Hypertension, *n* (%)	58 (43.9%)	38 (41.3%)	20 (50.0%)	0.355
Diabetes mellitus, *n* (%)	15 (11.4%)	12 (13.0%)	3 (7.5%)	0.533
Obesity, *n* (%)	20 (15.2%)	15 (16.3%)	5 (12.5%)	0.767
COPD, *n* (%)	5 (3.8%)	3 (3.3%)	2 (5.0%)	0.639
Coronary artery disease, *n* (%)	19 (14.4%)	10 (10.9%)	9 (22.5%)	0.080
Chronic kidney disease, *n* (%)	1 (0.8%)	1 (1.1%)	0 (0)	1.000
Chronic liver disease, *n* (%)	5 (3.8%)	3 (3.3%)	2 (5.0%)	0.639
Arrhythmia, *n* (%)	9 (6.8%)	6 (6.5%)	3 (7.5%)	1.000
Malignancy, *n* (%)	9 (6.8%)	6 (6.5%)	3 (7.5%)	1.000
**Laboratory findings**				
Lymphocyte count, × 10^9^/l	1.0 (0.6,1.5)	1.1 (0.7,1.6)	0.6 (0.4,1.1)	0.001
D-dimer, mg/l	1.1 (0.3, 3.0)	1.0 (0.4, 2.8)	1.6 (0.2, 4.3)	0.789
PT, s	13.7 (12.5, 15.0)	13.2 (12.4, 14.3)	13.9 (13.2, 15.3)	0.021
APTT, s	37.7 (33.1, 44.7)	36.8 (32.6, 42.1)	39.5 (36.7, 45.7)	0.013
CK-MB, U/l	10 (6, 15)	9 (5, 13)	14 (9, 30)	<0.001
hs-TNI, ng/l	4.1 (2.0, 30.2)	3.0 (1.5, 4.8)	85.6 (51.8, 262.1)	<0.001
BNP, pg/ml	62.4 (31.5, 164.2)	53.4 (29.3, 120.5)	130.5 (41.2, 449.0)	0.019
PaO_2_/FIO_2_, mm Hg	233.3 (153.5, 270.7)	236.4 (156.0, 272.4)	221.5 (144.7, 274.0)	0.559
CRP, mg/l	26.5 (3.8, 68.0)	15.8 (3.0, 52.6)	54.0 (18.4, 128.4)	0.001
PCT, ng/ml	0.09 (0.05, 0.21)	0.06 (0.04, 0.14)	0.23 (0.07, 0.39)	<0.001
IL-6, pg/ml	4.1 (2.0, 21.0)	3.9 (1.2, 7.8)	11.2 (2.9, 23.4)	0.039
**Treatments**				
Antiviral therapy, *n* (%)	122 (92.4%)	86 (93.5%)	36 (90.0%)	0.737
Antibiotic therapy, *n* (%)	98 (74.2%)	64 (69.6%)	34 (85.0%)	0.062
Glucocorticoid therapy, *n* (%)	57 (43.5%)	30 (32.6%)	27 (67.5%)	<0.001
Intravenous immune globulin, *n* (%)	49 (37.1%)	30 (32.6%)	19 (47.5%)	0.104
Anticoagulant therapy, *n* (%)	62 (47.0%)	41 (44.5%)	21 (52.5%)	0.401
Diuretics, *n* (%)	35 (26.5%)	21 (22.8%)	14 (35.9%)	0.145
Beta-blockers, *n* (%)	26 (19.7%)	19 (20.7%)	7 (17.5%)	0.676
Alpha-blockers, *n* (%)	2 (1.5%)	1 (1.1%)	1 (2.5%)	0.516
Calcium channel blockers, *n* (%)	40 (30.3%)	28 (30.4%)	12 (30.0%)	0.960
ACE inhibitor/ARB, *n* (%)	9 (6.9%)	7 (7.8%)	2 (5.0%)	0.840
Oxygen therapy, *n* (%)	117 (88.6%)	80 (87.0%)	37 (92.5%)	0.523
High-flow oxygen, *n* (%)	72 (55.0%)	45 (49.5%)	27 (67.5%)	0.056
Mechanical ventilation, *n* (%)	32 (24.2%)	15 (16.3%)	17 (42.5%)	0.001
IMV, *n* (%)	22 (16.7%)	10 (10.9%)	12 (30.0%)	0.007
NIMV, *n* (%)	10 (7.6%)	5 (5.4%)	5 (12.5%)	0.170
ICU admission, *n* (%)	25 (18.9%)	13 (14.1%)	12 (30.0%)	0.032
**Complications**				
Acute kidney injury, *n* (%)	20 (15.2%)	6 (6.5%)	14 (35.0%)	<0.001
ARDS, *n* (%)	49 (37.1%)	28 (30.4%)	21 (52.5%)	0.016
Shock, *n* (%)	1 (0.8%)	0 (0)	1 (2.5%)	0.303
**Prognosis**				
Discharge, *n* (%)	113 (85.6%)	88 (95.7%)	25 (62.5%)	<0.001
Death, *n* (%)	19 (14.4%)	4 (4.3%)	15 (37.5%)	<0.001

*Values are mean ± SD, n (%), median (interquartile range). ACE, angiotensin-converting enzyme; APTT, activated partial thromboplastin time; ARB, angiotensin II receptor blocker; ARDS, acute respiratory distress syndrome; BNP, B-type natriuretic peptide; CK-MB, creatine kinase muscle-brain; COPD, chronic obstructive pulmonary disease; COVID-19, coronavirus disease 2019; CRP, C-reactive protein; DBP, diastolic blood pressure; FIO_2_, fraction of inspiration oxygen; ICU, intensive care unit; IL-6, interleukin-6; IMV, invasive mechanical ventilation; hs-TNI, high-sensitivity troponin I; NIMV, non-invasive mechanical ventilation; PaO_2_, partial pressure of oxygen; PCT, procalcitonin; PT, prothrombin time; SBP, systolic blood pressure*.

### Echocardiographic Characteristics

LV GLS_4CH_ measurements were obtained in all patients. LV GLS measurements were feasible in 99 patients. LV GLS_4CH_ was strongly correlated with LV GLS (r = 0.93, *P* < 0.001). Furthermore, No significant difference between LV GLS_4CH_ and LV GLS was observed in our study (19.1 ± 2.9% vs. 19.1 ± 2.7%, *P* = 0.885) (Supplementary Figure 1). Therefore, we used LV GLS_4CH_ to assess the LV GLS in 132 patients with COVID-19 to obtain larger sample size. We consider it is reasonable to use LV GLS_4CH_ as a surrogate for LV GLS during the epidemic of COVID-19 to allow rapid image acquisition, improve feasibility in LV strain analysis and reduce contagion exposure duration to healthcare worker. Echocardiographic characteristics of COVID-19 patients are described in Table 2. Eleven patients had pericardial effusion. Patients with cardiac injury displayed lower TAPSE, LV GLS_4CH_, and RV FWLS, higher PASP, and higher proportion of pericardial effusion than those without cardiac injury. However, there was no significant difference in left and right heart size, LAS-peak, LV volumes, mass and diastolic function, LVEF, and moderate–severe MR and TR between these two groups. In addition, LV GLS_4CH_ and RV FWLS was lower in patients with ARDS than those without (18.1 ± 2.7% vs. 19.7 ± 3.3%, *P* = 0.004; 21.0 ± 4.9% vs. 23.7 ± 4.7%, *P* = 0.003; respectively), whereas LAS-peak did not differ between patients with ARDS and without (33.0 ± 8.2 % vs. 33.4 ± 8.2%, *P* = 0.793).

**Table 2:** Echocardiographic characteristics of patients with COVID-19 according to acute cardiac injury.

**Variables**	**All patients (*n* = 132)**	**Without cardiac injury (*n =* 92)**	**With cardiac injury (*n =* 40)**	***P-*value**
**Left heart**				
LA dimension, mm	34.3 ± 5.4	34.4 ± 5.3	34.1 ± 5.7	0.728
LV dimension, mm	45.5 ± 4.9	45.6 ± 4.8	45.3 ± 4.9	0.715
IVS, mm	9.6 ± 1.3	9.5 ± 1.4	9.9 ± 1.1	0.183
PW, mm	9.2 (8.3, 9.9)	9.0 (8.1, 9.8)	9.6 (8.9, 10.4)	0.018
LVM, g	143.2 (116.0, 168.5)	143.1 (117.9, 168.6)	154.4 (114.9, 168.6)	0.707
DT, ms	204.4 ± 53.7	203 ± 54.6	207 ± 50.5	0.686
E/A ratio	0.8 (0.7,1.1)	0.8 (0.7,1.1)	0.8 (0.7,1.1)	0.884
E/e′ ratio	8.4 (6.8,10.6)	8.9 ± 3.1	9.4 ± 3.4	0.453
LVEDVI, ml/m^2^	52.2 ± 16.1	53.3 ± 14.8	49.5 ± 19.4	0.349
LVESVI, ml/m^2^	19.6 ± 7.5	20.2 ± 7.0	18.1 ± 8.8	0.256
LVEF, %	62.8 ± 6.9	62.7 ± 7.4	63.2 ± 5.8	0.735
LV GLS_4CH_, %	18.9 (16.8, 20.9)	19.1 (17.1, 20.9)	17.3 (15.8, 20.4)	0.017
LAS-peak, %	33.7 ± 7.6	34.1 ± 8.0	33.0 ± 6.7	0.489
Moderate-severe MR, *n* (%)	2 (1.5%)	0 (0)	2 (5.0%)	0.090
**Right heart**				
RA dimension, mm	35.5 ± 4.6	35.2 ± 4.4	36.6 ± 5.1	0.126
RV dimension, mm	33.9 ± 4.4	33.6 ± 4.3	34.7 ± 4.7	0.250
TAPSE, mm	22.2 ± 3.8	22.8 ± 3.8	20.8 ± 3.2	0.005
RVFAC, %	46.9 ± 6.6	47.6 ± 6.3	45.3 ± 7.1	0.066
S′, cm/s	13.3 (11.9,15.0)	14.0 (12.0,15.0)	13.0 (11.0,15.0)	0.361
RV FWLS, %	22.8 ± 4.9	23.5 ± 5.2	21.1 ± 3.8	0.009
Moderate-severe TR, *n* (%)	4 (3.0%)	2 (2.2%)	2 (5.0%)	0.584
PASP, mm Hg	33 (24,47)	28 (23,43)	41 (30,54)	0.007
Pericardial effusion, *n* (%)	11 (8.3%)	4 (4.3%)	7 (17.5%)	0.030

*Values are mean ± SD, n (%), median (interquartile range). COVID-19, coronavirus disease 2019; DT, peak E deceleration time of mitral inflow; IVS., interventricular septum; LA, left atrial; LAS, left atrial strain; LV, left ventricular; LV GLS_4CH_, left ventricular global longitudinal strain derived from the apical four-chamber view; LVEDVI, left ventricular end diastolic volume index; LVEF, left ventricular ejection fraction; LVESVI, left ventricular end systolic volume index; LVM, left ventricular mass; MR, mitral regurgitation; PASP, pulmonary artery systolic pressure; PW, posterior wall of left ventricle; RA, right atrial; RV, right ventricular; RV FWLS, right ventricular free wall longitudinal strain; RVFAC, RV fractional area change; SBP, systolic blood pressure; TAPSE, tricuspid annular plane systolic excursion; TR, tricuspid regurgitation. LV GLS_4CH_ and RV FWLS values are absolute values*.

According to the seventh version of the guidelines on the Diagnosis and Treatment of COVID-19 by the National Health Commission, COVID-19 severity is classified as mild, moderate, severe and critical types (10). There were 50 moderate, 35 severe, and 47 critical patients in our study. Our results revealed that critical group had decreased LV GLS_4CH_, RV FWLS, and TAPSE, elevated PASP, higher proportion of moderate-severe TR, and higher mortality compared with moderate and severe groups. There was no significant difference in LVEF, LAS-peak, RVFAC and S′ among the moderate, severe, and critical groups (Table 3).

**Table 3:** Clinical and echocardiographic characteristics of patients with COVID-19 according to disease severity.

			**Disease severity**		
**Variables**	**All patients (*n =* 132)**	**Moderate (*n =* 50)**	**Severe (*n =* 35)**	**Critical (*n =* 47)**	***p*-value**
**Clinical characteristics**					
Age, years	61 ± 13	59 ± 12	62 ± 15	63 ± 12	0.241
Male, *n* (%)	68 (51.5%)	20 (40.0%)	19 (54.3%)	29 (61.7%)	0.093
Body mass index, kg/m^2^	23.6 ± 2.9	23.6 ± 2.6	23.4 ± 3.0	23.9 ± 3.2	0.810
Heart rate, beats/min	91 ± 17	90 ± 16	88 ± 15	94 ± 19	0.216
Respiratory rate, breaths/min	25 ± 6	24 ± 6	24 ± 5	25 ± 7	0.707
SBP, mm Hg	134 ± 18	134 ± 17	133 ± 20	131 ± 17	0.742
DBP, mm Hg	81 ± 12	82 ± 10	81 ± 14	80 ± 12	0.767
**Left heart**					
LA dimension, mm	34.3 ± 5.4	34.3 ± 4.9	35.2 ± 4.7	34.2 ± 6.5	0.466
LV dimension, mm	45.5 ± 4.9	44.9 ± 4.6	46.4 ± 4.8	45.8 ± 5.1	0.526
IVS, mm	9.6 ± 1.3	9.7 ± 1.1	9.6 ± 1.9	9.6 ± 1.0	0.629
PW, mm	8.8 ± 1.9	9.2 ± 1.1	8.2 ± 2.7*	9.0 ± 1.7	0.049
LVM, g	144.3 ± 36.1	143.7 ± 35.6	148.0 ± 40.9	144.7 ± 33.6	0.861
DT, ms	204.4 ± 53.7	209.3 ± 60.4	190.0 ± 48.8	209.8 ± 46.5	0.176
E/A ratio	0.8 (0.7, 1.1)	0.8 (0.7, 1.1)	0.9 (0.7, 1.3)	0.8 (0.7, 1.0)	0.515
E/e′ ratio	8.4 (6.8, 10.6)	8.4 (6.5, 10.0)	8.8 (6.3, 10.6)	8.0 (6.9, 10.9)	0.891
LVEDVI, ml/m^2^	52.2 ± 16.1	55.0 ± 15.6	53.0 ± 17.5	48.9 ± 14.4	0.321
LVESVI, ml/m^2^	17.5 (15.2, 22.9)	17.5 (15.2, 22.8)	18.6 (15.8, 24.2)	16.2 (13.8, 23.1)	0.724
LVEF, %	62.8 ± 6.9	63.4 ± 7.7	62.5 ± 7.4	62.9 ± 5.8	0.882
LV GLS_4CH_, %	18.9 (16.8, 20.9)	20.1 (18.2, 22.0)	19.0 (17.3, 21.9)	17.0 (15.7, 18.6)^*#^	<0.001
LAS-peak, %	33.7 ± 7.6	31.9 ± 7.9	35.8 ± 8.1	32.0 ± 10.2	0.146
Moderate-severe MR, *n* (%)	2 (1.5%)	0 (0)	0 (0)	2 (4.3%)	0.090
**Right heart**					
RA dimension, mm	35.5 ± 4.6	34.7 ± 4.9	36.4 ± 3.4	35.9 ± 5.1	0.209
RV dimension, mm	34.0 (30.6, 36.4)	33.3 (29.8, 35.8)	34.4 (32.4, 36.6)	34.0 (30.7, 36.6)	0.485
TAPSE, mm	22.2 ± 3.8	22.8 ± 3.8	22.6 ± 3.5	20.8 ± 3.7^*#^	0.019
RVFAC, %	47.2 (41.6, 51.2)	49.3 (42.0, 52.2)	45.5 (40.8, 51.2)	46.9 (40.9, 50.3)	0.255
S′, cm/s	13.3 (11.9, 15.0)	14.0 (12.0, 15.0)	14.0 (12.0, 16.4)	12.9 (11.0, 15.0)	0.301
RV FWLS, %	22.7 (19.2, 25.6)	23.9 (20.1, 26.2)	23.6 (19.8, 26.1)	20.2 (18.1, 24.0)^*#^	0.015
Moderate-severe TR, *n* (%)	4 (3.0%)	0 (0)	0 (0)	4 (8.5%)	0.016
PASP, mm Hg	36 ± 14	30 ± 11	39 ± 14*	39 ± 15*	0.037
**Prognosis**					
Discharge, *n* (%)	113 (85.6%)	50 (100.0%)	33 (97.1%)	30 (62.5%)^*#^	<0.001
Death, *n* (%)	19 (14.4%)	0 (0)	1 (2.9%)	18 (37.5%)^*#^	<0.001

Values are mean ± SD, n (%), median (interquartile range). *P < 0.05, severe or critical vs. moderate; ^#^P < 0.05, critical vs. severe. COVID-19, coronavirus disease 2019; DBP, diastolic blood pressure; DT, peak E deceleration time of mitral inflow; IVS., interventricular septum; LA, left atrial; LAS, left atrial strain; LV, left ventricular; LV GLS_4CH_, left ventricular global longitudinal strain derived from the apical four-chamber view; LVEDVI, left ventricular end diastolic volume index; LVEF, left ventricular ejection fraction; LVESVI, left ventricular end systolic volume index; LVM, left ventricular mass; MR, mitral regurgitation; PASP, pulmonary artery systolic pressure; PW, posterior wall of left ventricle; RA, right atrial; RV, right ventricular; RV FWLS, right ventricular free wall longitudinal strain; RVFAC, RV fractional area change; SBP, systolic blood pressure; TAPSE, tricuspid annular plane systolic excursion; TR, tricuspid regurgitation. LV GLS_4CH_ and RV FWLS values are absolute values.

At the time of the echocardiographic examinations, 32 patients were intubated. 117 (88.6%) patients were in oxygen therapy. 72 (55.0%) patients were treated with high-flow oxygen. Compared with patients who did not require mechanical ventilation, those who required mechanical ventilation had impaired LV GLS_4CH_, RV FWLS, TAPSE and RVFAC, and elevated PASP, whereas LVEF and LAS-peak were not different between these two groups (Supplemental Table 1).

During hospitalization, 19 patients died. Compared with survivors, non-survivors displayed dilated right heart chamber, impaired TAPSE, RVFAC, S′, RV FWLS and LV GLS_4CH_, higher proportion of moderate–severe MR and TR, and higher PASP. In contrast, left heart chamber dimension, LAS-peak, LV wall thickness, mass and diastolic function, and LVEF were similar between survivors and non-survivors (Table 4).

**Table 4:** Clinical and echocardiographic characteristics of survivors and non-survivors with COVID-19.

**Variables**	**All patients (*n* = 132)**	**Survivor (*n =* 113)**	**Non-survivor (*n =* 19)**	***P-*value**
**Clinical characteristics**				
Age, years	61 ± 13	61 ± 13	64 ± 13	0.556
Male, *n* (%)	68 (51.5%)	54 (47.8%)	14 (73.7%)	0.037
Body mass index, kg/m^2^	23.6 ± 2.9	23.6 ± 2.9	23.7 ± 3.5	0.701
Heart rate, beats/min	91 ± 17	90 ± 16	96 ± 22	0.092
Respiratory rate, breaths/min	25 ± 6	24 ± 5	28 ± 8	0.059
SBP, mm Hg	134 ± 18	133 ± 18	134 ± 16	0.914
DBP, mm Hg	81 ± 12	81 ± 12	78 ± 14	0.245
**Left heart**				
LA dimension, mm	34.2 (31.7, 37.0)	34.2 (31.6, 36.9)	35.7 (31.7, 37.7)	0.602
LV dimension, mm	45.8 (42.3, 49.0)	45.9 (42.3, 49.3)	45.8 (41.7, 48.4)	0.751
IVS, mm	9.6 (8.9, 10.4)	9.6 (9.0, 10.4)	9.6 (9.2, 10.1)	0.962
PW, mm	9.2 (8.3, 9.9)	9.0 (8.1, 9.9)	9.4 (8.9, 9.7)	0.350
LVM, g	143.2 (116.0, 168.5)	143.0 (121.5, 169.3)	148.7 (114.1, 170.4)	0.824
DT, ms	202.0 (163.9, 235.0)	206.5 (162.5, 239.0)	195.0 (164.0, 222.0)	0.488
E/A ratio	0.8 (0.7, 1.1)	0.8 (0.7, 1.1)	0.9 (0.7, 1.4)	0.218
E/e′ ratio	8.4 (6.8, 10.6)	8.1 (6.8, 10.1)	8.9 (7.1, 11.7)	0.274
LVEDVI, ml/m^2^	49.8 (39.2, 59.3)	51.1 (41.1, 63.0)	38.9 (33.1, 38.9)	0.043
LVESVI, ml/m^2^	17.5 (15.2, 22.9)	18.2 (15.5, 23.3)	14.2 (10.6, 23.5)	0.077
LVEF, %	63.2 (59.1, 68.0)	63.3 (59.0, 68.0)	63.4 (59.9, 67.8)	0.635
LV GLS_4CH_, %	18.9 (16.8, 20.9)	19.3 (17.3, 21.6)	16.0 (14.7, 16.9)	<0.001
LAS-peak, %	33.7 (27.6, 37.9)	33.4 (27.0, 39.5)	30.2 (27.1, 36.7)	0.155
Moderate-severe MR, *n* (%)	2 (1.5%)	0 (0)	2 (10.5%)	0.020
**Right heart**				
RA dimension, mm	35.7 (32.4, 38.2)	35.1 (32.1, 37.7)	37.6 (34.2, 39.1)	0.030
RV dimension, mm	34.0 (30.6, 36.4)	33.2 (30.3, 35.7)	35.8 (32.0, 41.0)	0.022
TAPSE, mm	22.2 (19.1, 25.2)	22.3 (20.2, 25.4)	19.0 (17.1, 21.1)	0.001
RVFAC, %	47.2 (41.6, 51.2)	48.2 (42.0, 52.0)	43.2 (37.8, 49.0)	0.008
S′, cm/s	13.3 (11.9, 15.0)	14.0 (12.0, 15.7)	11.7 (10.0, 14.7)	0.017
RV FWLS, %	22.7 (19.2, 25.6)	23.6 (20.0, 26.1)	18.0 (17.3, 20.6)	<0.001
Moderate-severe TR, *n* (%)	4 (3.0%)	2 (1.8%)	3 (15.8%)	0.018
PASP, mm Hg	33 (24, 47)	31 (24, 47)	47 (32, 60)	0.041

*Values are mean ± SD, n (%), median (interquartile range). COVID-19, coronavirus disease 2019; DBP, diastolic blood pressure; DT, peak E deceleration time of mitral inflow; IVS., interventricular septum; LA, left atrial; LAS, left atrial strain; LV, left ventricular; LV GLS_4CH_, left ventricular global longitudinal strain derived from the apical four-chamber view; LVEDVI, left ventricular end diastolic volume index; LVEF, left ventricular ejection fraction; LVESVI, left ventricular end systolic volume index; LVM, left ventricular mass; MR, mitral regurgitation; PASP, pulmonary artery systolic pressure; PW, posterior wall of left ventricle; RA, right atrial; RV, right ventricular; RV FWLS, right ventricular free wall longitudinal strain; RVFAC, RV fractional area change; SBP, systolic blood pressure; TAPSE, tricuspid annular plane systolic excursion; TR, tricuspid regurgitation. LV GLS_4CH_ and RV FWLS values are absolute values*.

### Follow-Up Study in COVID-19 Patients Who Were Alive

**Figure 2:**
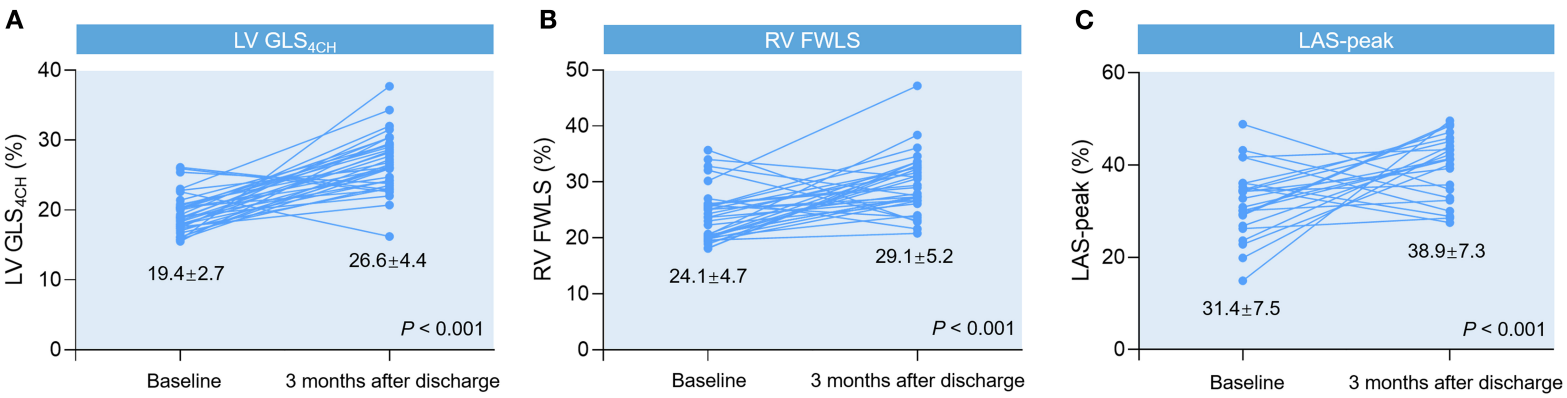
Spaghetti plots of LV GLS_4CH_ **(A)**, RV FWLS **(B)**, and LAS-peak **(C)** in patients with COVID-19 at 3-month follow-up after discharge compared with baseline values.

Forty-six survivors were followed up at 3 months after discharge (Table 5). We observed significant improvements in LV GLS_4CH_, RV FWLS, and LAS-peak (Figure 2), and a decrease in PASP in recovered patients, whereas LVEF and conventional RV function parameters (TAPSE, S′ and RVFAC) were not different from the baseline values (*P* > 0.05).

**Table 5:** Clinical and echocardiographic characteristics of patients with COVID-19 three months after discharge.

**Variables**	**Baseline (*n =* 46)**	**3 months after discharge (*n =* 46)**	***P-*value**
**Clinical characteristics**			
Age, years	59 ± 13		
Male, *n* (%)	18 (39.1%)		
Body mass index, kg/m^2^	23.3 ± 3.0		
Heart rate, beats/min	94 ± 18	80 ± 12	<0.001
Respiratory rate, breaths/min	24 ± 5	21 ± 2	0.010
SBP, mm Hg	137 ± 19	134 ± 16	0.509
DBP, mm Hg	82 ± 10	86 ± 10	0.075
**Laboratory findings**			
Lymphocyte count, × 10^9^/l	1.0 (0.5, 1.4)	1.9 (1.5, 2.5)	<0.001
D-dimer, mg/l	0.59 (0.16, 1.92)	0.34 (0.24, 0.55)	0.096
hs-TNI, ng/l	4.4 (1.7, 60.8)	1.4 (0.3, 2.6)	0.001
CRP, mg/l	35.8 (4.3, 75.1)	1.0 (0.6, 3.1)	<0.001
**Left heart**			
LA dimension, mm	36.7 ± 4.4	36.5 ± 5.2	0.864
LV dimension, mm	46.4 ± 4.7	46.1 ± 3.6	0.786
IVS, mm	9.5 ± 1.2	8.8 ± 1.2	0.007
PW, mm	8.7 ± 1.6	8.6 ± 2.1	0.813
LVM, g	136.1 ± 37.7	134.2 ± 34.9	0.811
DT, ms	195 ± 52	199 ± 45	0.751
E/A ratio	0.9 ± 0.4	1.0 ± 0.7	0.255
E/e′ ratio	8.8 ± 2.9	8.1 ± 3.6	0.360
LVEDVI, ml/m^2^	56.5 ± 18.9	56.0 ± 17.1	0.916
LVESVI, ml/m^2^	21.6 ± 8.9	20.3 ± 7.3	0.571
LVEF, %	62.1 ± 8.2	63.1 ± 8.0	0.613
LV GLS_4CH_, %	19.4 ± 2.7	26.6 ± 4.4	<0.001
LAS-peak, %	31.4 ± 7.5	38.9 ± 7.3	<0.001
**Right heart**			
RA dimension, mm	35.7 ± 3.5	33.9 ± 3.4	0.023
RV dimension, mm	33.5 ± 3.2	33.3 ± 3.4	0.804
TAPSE, mm	22.8 ± 3.6	23.5 ± 8.3	0.636
RVFAC, %	48.8 ± 7.1	49.6 ± 10.0	0.699
S′, cm/s	14.0 ± 2.6	13.8 ± 2.4	0.722
RV FWLS, %	24.1 ± 4.7	29.1 ± 5.2	<0.001
PASP, mm Hg	36 ± 10	27 ± 7	0.026

*Values are mean ± SD, n (%), median (interquartile range). COVID-19, coronavirus disease 2019; DBP, diastolic blood pressure; DT, peak E deceleration time of mitral inflow; IVS., interventricular septum; LA, left atrial; LAS, left atrial strain; LV, left ventricular; LV GLS_4CH_, left ventricular global longitudinal strain derived from the apical four-chamber view; LVEDVI, left ventricular end diastolic volume index; LVEF, left ventricular ejection fraction; LVESVI, left ventricular end systolic volume index; LVM, left ventricular mass; PASP, pulmonary artery systolic pressure; PW, posterior wall of left ventricle; RA, right atrial; RV, right ventricular; RV FWLS, right ventricular free wall longitudinal strain; RVFAC, RV fractional area change; SBP, systolic blood pressure; TAPSE, tricuspid annular plane systolic excursion. LV GLS_4CH_ and RV FWLS values are absolute values*.

### Correlation of Biventricular Function With Cardiac Injury and Inflammatory Marker

A decrease in LV GLS_4CH_ weakly correlated with decreased lymphocyte count (r = 0.37, *P* < 0.001), and elevated levels of CRP (r = −0.39, *P* < 0.001), PCT (r = −0.31, *P* = 0.001), IL-6 (r = −0.28, *P* = 0.041), CK-MB (r = −0.17, *P* = 0.044), hs-TNI (r = −0.30, *P* = 0.001), D-dimer (r = −0.24, *P* = 0.012) and APTT (r = −0.26, *P* = 0.003) (Supplementary Figure 2). A reduction in RV FWLS had weak correlations with higher levels of CRP (r = −0.29, *P* = 0.001), PCT (r = −0.33, *P* = 0.001), CK-MB (r = −0.21, *P* = 0.018), hs-TNI (r = −0.43, *P* < 0.001), APTT (r = −0.26, *P* = 0.003), and PT (r = −0.30, *P* = 0.001) (Supplementary Figure 3). Additionally, decreased LV GLS_4CH_ and RV FWLS were also related to the presence of pericardial effusion (r = −0.217, *P* = 0.012; r = −0.339, *P* < 0.001, respectively). In contrast, LAS-peak and LVEF had no significant correlation with biomarkers levels of inflammation, coagulopathy, and cardiac injury (*P* > 0.05 for all).

### Predictors of Mortality in Patients With COVID-19

A univariate Cox regression analysis showed that elevated level of hs-TNI, ARDS, LV GLS_4CH_, RV FWLS, TAPSE, and RVFAC were associated with higher risk of mortality (Table 6). Whereas, LAS-peak, LVEF and S′ were not predictive of death. The multivariate Cox analysis models revealed that hs-TNI elevation and ARDS continued to be of prognostic significance. LV GLS_4CH_ [hazard ratio [HR]: 1.41, 95% confidence intervals [CI]: 1.08-1.84; *P* = 0.011], RV FWLS (HR: 1.29, 95% CI: 1.09-1.52; *P* = *0.0*03), TAPSE (HR: 0.82, 95% CI: 0.69-0.98; *P* = 0.031), and RVFAC (HR: 0.92, 95% CI: 0.85-0.99; *P* = 0.032) were independent predictive of higher risk of death. The Cox models using LV GLS_4CH_ (AIC = 131) or RV FWLS (AIC = 122) were observed to predict higher mortality more accurately than that with TAPSE (AIC = 134), RVFAC (AIC = 134) or traditional risk model (AIC = 138) (Table 6).

**Table 6:** Predictors of mortality in patients with COVID-19 by cox proportional hazard model.

	**Univariate Cox regression**		**Model 1 ARDS + hs-TNI**		**Model 2 ARDS + hs-TNI + LV GLS**_****4CH****_		**Model 3 ARDS + hs-TNI + TAPSE**		**Model 4 ARDS + hs-TNI + RVFAC**		**Model 5 ARDS + hs-TNI + RV FWLS**	
	**HR (95% CI)**	***p-*value**	**HR (95% CI)**	***p-*value**	**HR (95% CI)**	***p-*value**	**HR (95% CI)**	***p-*value**	**HR (95% CI)**	***p-*value**	**HR (95% CI)**	***p-*value**
Age, years	1.02 (0.99, 1.06)	0.354										
Male (yes vs. no)	3.06 (1.01, 9.22)	0.048										
Hypertension (yes vs. no)	2.58 (0.98, 6.79)	0.055										
Diabetes mellitus (yes vs. no)	0.38 (0.05, 2.86)	0.349										
Obesity, *n* (%)	0.88 (0.25, 3.04)	0.837										
Coronary artery disease (yes vs. no)	1.49 (0.53, 4.18)	0.447										
Malignancy (yes vs. no)	1.99 (0.46, 8.65)	0.359										
Arrhythmia (yes vs. no)	1.09 (0.25, 4.79)	0.909										
ARDS (yes vs. no)	7.50 (2.18, 25.80)	0.001	5.52 (1.59, 19.22)	0.007	4.27 (1.20, 15.21)	0.025	5.43 (1.50, 19.65)	0.010	5.90 (1.68, 20.71)	0.006	3.77 (1.04, 13.67)	0.044
Elevated CK-MB (yes vs. no)	0.20 (0.03, 1.49)	0.116										
Elevated hs-TNI (yes vs. no)	8.13 (2.69, 24.51)	<0.001	6.23 (2.04, 19.00)	0.001	3.53 (1.06, 11.80)	0.041	3.70 (1.12, 12.23)	0.032	4.36 (1.36, 13.95)	0.013	4.28 (1.36, 13.47)	0.013
Elevated BNP (yes vs. no)	0.70 (0.57, 4.17)	0.397										
PaO_2_:FIO_2_, mmHg	1.00 (0.98, 1.01)	0.599										
Mechanical ventilation (yes vs. no)	2.20 (0.89, 5.42)	0.088										
ACE inhibitor/ARB (yes vs. no)	0.59 (0.08, 4.45)	0.610										
Pericardial effusion (yes vs. no)	1.93 (0.56, 6.68)	0.299										
E/e′ ratio	1.02 (0.90, 1.16)	0.794										
LVEDVI, ml/m^2^	0.95 (0.90, 1.00)	0.070										
LVESVI, ml/m^2^	0.90 (0.84, 1.03)	0.163										
LVM, g	1.00 (0.99, 1.01)	0.771										
LVEF, %	1.02 (0.95, 1.10)	0.607										
LV GLS_4CH_, %	1.70 (1.30, 2.23)	<0.001			1.41 (1.08, 1.84)	0.011						
LAS-peak, %	0.96 (0.90, 1.03)	0.217										
TAPSE, mm	0.81 (0.70, 0.93)	0.003					0.82 (0.69, 0.98)	0.031				
RVFAC, %	0.89 (0.82, 0.97)	0.007							0.92 (0.85, 0.99)	0.032		
S′, cm/s	0.83 (0.68, 1.01)	0.058										
RV FWLS, %	1.32 (1.15, 1.50)	<0.001									1.29 (1.09, 1.52)	0.003
AIC	/	/	138		131		134		134		122	

*ACE, angiotensin-converting enzyme; AIC, Akaike Information Criterion; ARB, angiotensin II receptor blockers; ARDS, acute respiratory distress syndrome; BNP, B-type natriuretic peptide; CI, confidence interval; CK-MB, creatine kinase muscle-brain; COVID-19, coronavirus disease 2019; FIO_2_, fraction of inspiration oxygen; HR, hazard ratio; hs-TNI, high-sensitivity troponin I; LAS, left atrial strain; LV GLS_4CH_, left ventricular global longitudinal strain derived from the apical four-chamber view; LVEDVI, left ventricular end diastolic volume index; LVEF, left ventricular ejection fraction; LVESVI, left ventricular end systolic volume index; LVM, left ventricular mass; PaO_2_, partial pressure of oxygen; RV FWLS, right ventricular free wall longitudinal strain; RVFAC, right ventricular fractional area change; TAPSE, tricuspid annular plane systolic excursion. CK-MB ≥ 25 U/l was defined as elevated CK-MB; hs-TNI ≥ 26.2 ng/ml was defined as elevated hs-TNI; BNP ≥ 100 pg/ml was defined as elevated BNP*.

**Figure 3:**
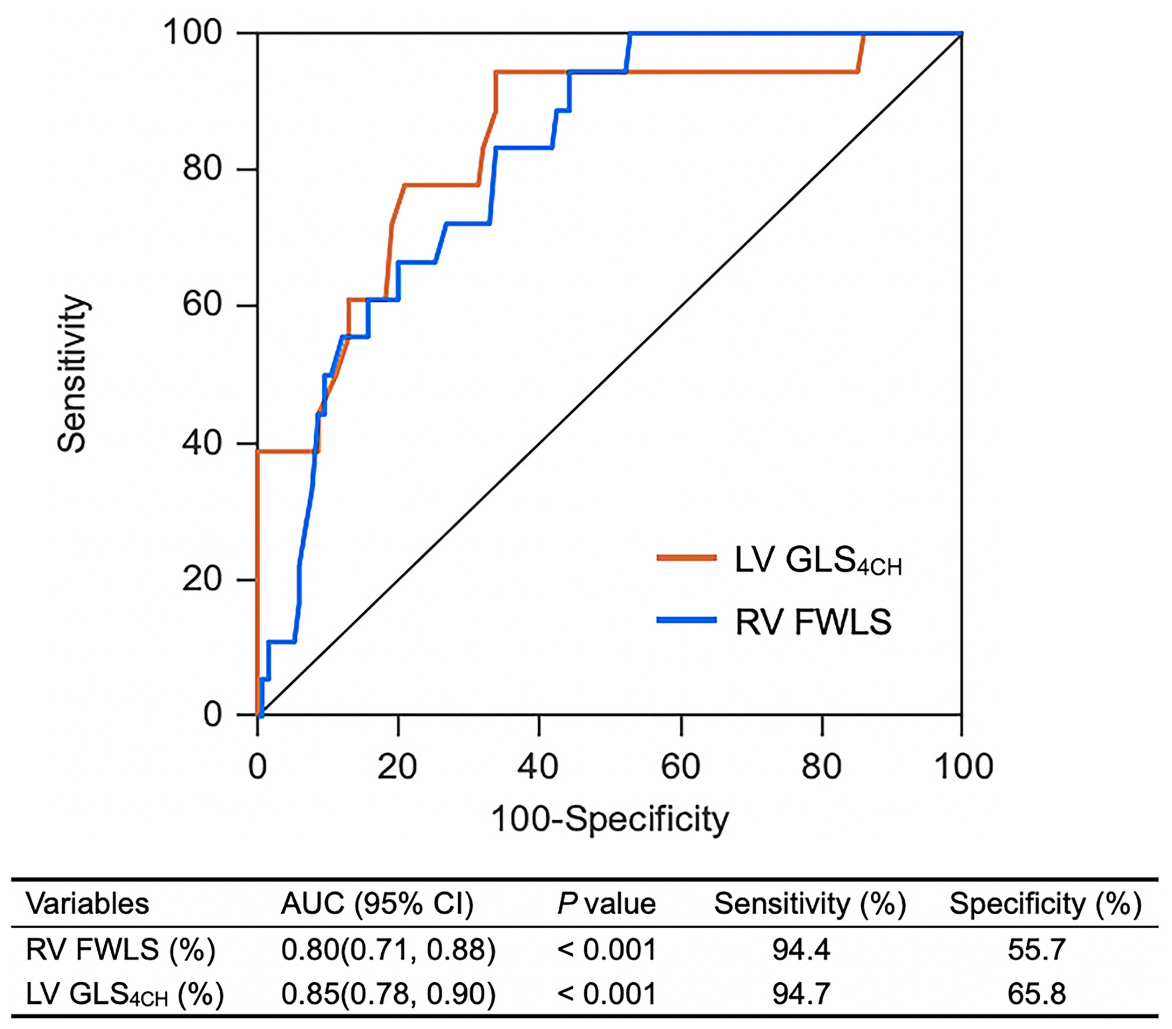
ROC curves of biventricular longitudinal strain for adverse clinical outcome. LV GLS_4CH_, left ventricular global longitudinal strain from the apical four-chamber view; RV FWLS, Right ventricular free wall longitudinal strain.

LAS-peak, LV GLS_4CH_, RV FWLS, conventional RV function parameters and LVEF were entered into ROC analysis to estimate probability of in-hospital death. Impaired LV GLS_4CH_ and RV FWLS were associated with higher mortality (Figure 3). Areas under the curve were 0.85 for LV GLS_4CH_ and 0.80 for RV FWLS. The optimal cutoff value of LV GLS_4CH_ for detection of increased mortality was −17.9% with sensitivity of 94.7% and specificity of 65.8%. The best cutoff value of RV FWLS for identification of death was −22.9% (sensitivity, 94.4%; specificity, 55.7%).

**Figure 4:**
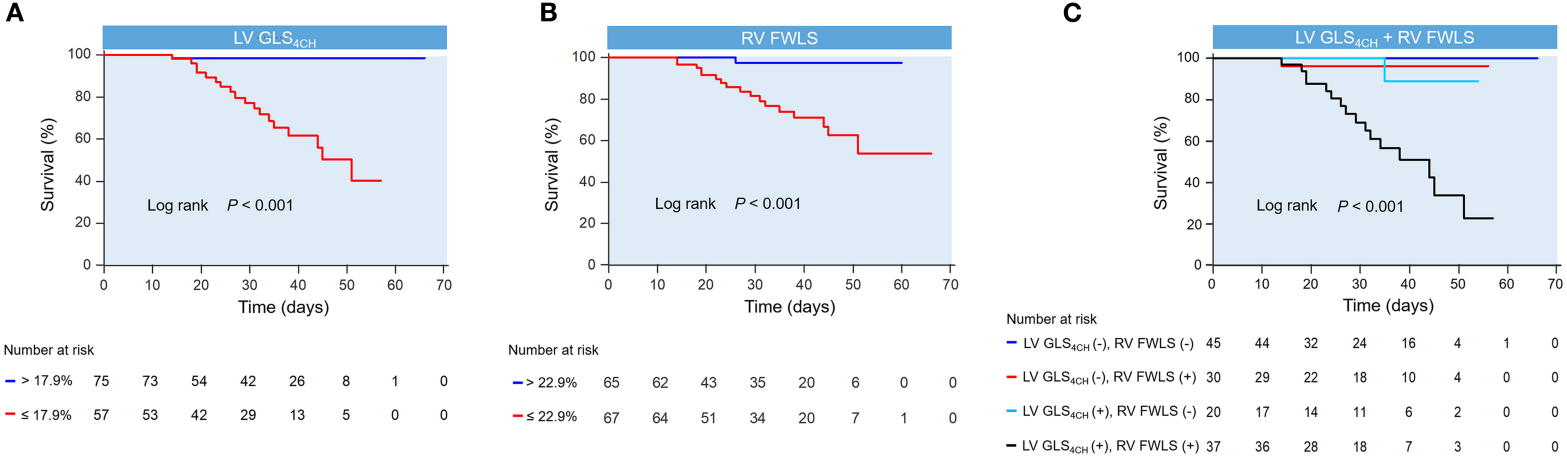
Kaplan-meier survival curves showing the association of biventricular longitudinal strain and higher mortality. Kaplan-Meier curves in COVID-19 patients stratified by the cutoff value of LV GLS_4CH_ **(A)** and RV FWLS **(B)**. **(C)** Kaplan-Meier curves reveal that COVID-19 patients below cutoff LV GLS_4CH_ and RV FWLS have the highest mortality. LV GLS_4CH_ and RV FWLS values are absolute values. LV GLS_4CH_ (+), below cutoff LV GLS_4CH_; LV GLS_4CH_ (–), above cutoff LV GLS_4CH_; RV FWLS (+), below cutoff RV FWLS; RV FWLS (–), above cutoff RV FWLS.

**Figure 5:**
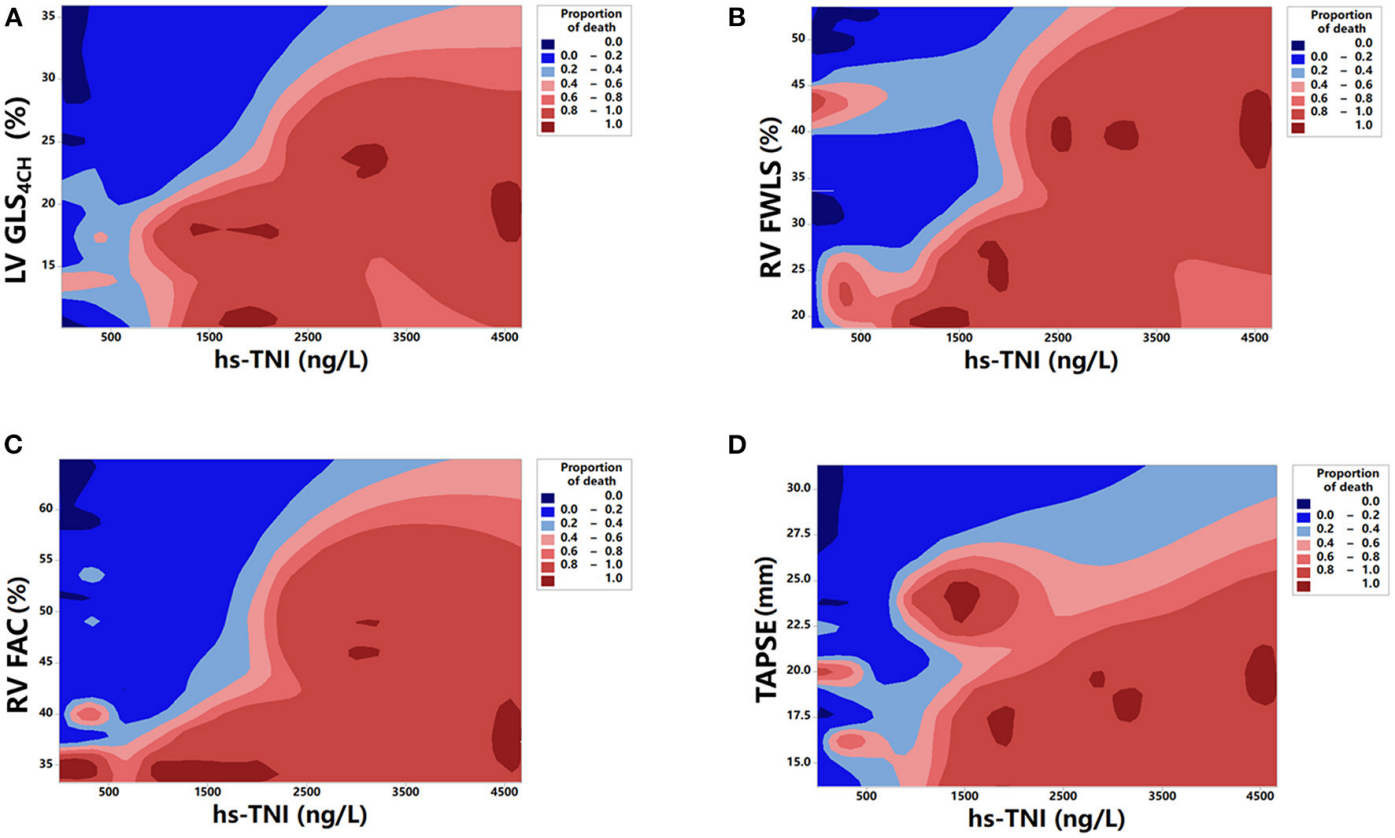
Contour plot of survival probability in hospitalized patients with COVID-19. Decreased LV GLS_4CH_ **(A)**, RV FWLS **(B)**, RV FAC **(C)**, and TAPSE **(D)** are associated with higher mortality, which is pronounced in patients with higher levels of hs-TNI.

Kaplan-Meier survival curves of biventricular longitudinal strain for mortality are presented in Figure 4. When stratified by cutoff values, LV GLS_4CH_ lower than 17.9 % or RV FWLS lower than 22.9% were associated with higher mortality (*P* < 0.001) (Figures 4A,B). Patients with below cutoff LV GLS_4CH_ and RV FWLS had the worst prognosis compared those with above cutoff LV GLS_4CH_ and RV FWLS (Figure 4C). To determine the relationship between levels of hs-TNI, cardiac function parameters and mortality, a contour plot was performed. Our findings revealed that decreased LV GLS_4CH_, RV FWLS, RVFAC, and TAPSE were associated with increased death, which was pronounced in patients with higher levels of hs-TNI (Figure 5).

### Reproducibility

The intraobserver and interobserver reproducibility of LV GLS_4CH_, RV FWLS and LAS-peak are summarized in Supplemental Table 2. The intraobserver and interobserver reproducibility of LV GLS_4CH_, RV FWLS, and LAS-peak were high.

## Discussion

To the best of our knowledge, our study is the first to systematically assess cardiac structure and function in COVID-19 patients using both conventional echocardiography and 2D-STE. This study demonstrates that patients with cardiac injury had higher levels of coagulopathy and inflammatory biomarkers, higher incidence of complications, more treatment with mechanical ventilation, higher mortality, and lower LV GLS_4CH_ and RV FWLS than those without cardiac injury. Compared with survivors, non-survivors displayed reduced biventricular longitudinal strain, and comparable LVEF. At a 3-month follow-up after discharge, we identify that biventricular longitudinal strain can track clinical improvement in the convalescent phase. Importantly, LV GLS_4CH_ and RV FWLS are powerful predictors of higher mortality in patients with COVID-19. Therefore, biventricular longitudinal strain may be essential for risk stratification and serial follow-up in patients with COVID-19.

### Biventricular Function in Patients With COVID-19

SARS-CoV-2 are known to result in the acute and chronic damage of the cardiovascular system (11, 12). Although several recent studies have demonstrated that 5.2%-23% patients with COVID-19 suffered myocardial injury from the infection (12–14), there are limited echocardiographic data regarding the cardiac abnormalities. Prior report highlights the significance of assessing cardiac function of hospitalized COVID-19 patients (15).

Despite the importance and extensive use of LVEF in routine clinical practice, there are several limitations of its application. First, it depends on geometric assumptions and loading conditions. Moreover, it could not reflect myocardial contractility (16). Finally, LVEF may have considerable inter- and intra-observer variability. Accordingly, LVEF may not be an optimal index to detect myocardial impairment. Novel, more sensitive indices for cardiac dysfunction at an earlier stage are required. Recently, LV and RV longitudinal strain have been recommended as sensitive and early indicators of subclinical cardiac dysfunction (17). They are measurements of myocardial deformation, and objective parameters with excellent reproducibility and high feasibility. We previously found that COVID-19 patients had impaired RV FWLS (18). However, there are no data regarding the use of LV GLS_4CH_ in patients with COVID-19. In the present study, we identified that COVID-19 patients exhibited significantly impaired LV GLS_4CH_ and RV FWLS, while no difference was found in LVEF. Moreover, impaired biventricular longitudinal strain appeared to be worse in critically ill patients or those who required mechanical ventilation therapy. These findings are in agreement with the study of SARS, which revealed that the diminished LV performance was worse in patients who needed treatment with mechanical ventilation (19). In a study of 28 patients with acute myocarditis, reduced LV GLS correlated with the amount of oedema, and added important information on the diagnosis and degree of myocardial dysfunction, especially in patients with preserved LVEF (20). Recently, there are increasing data regarding the cardiac impairment in patients diagnosed with COVID-19 infection (21–24). The mechanisms of cardiac injury are uncertain but likely involve direct viral injury, aggravation of a systemic inflammatory response, hypoxemia, destabilized coronary plaques and microthrombogenesis (25). Consistent with this postulation, the correlations of diminished LV GLS_4CH_ and RV FWLS with elevated biomarkers levels of inflammation, coagulopathy, and cardiac injury were observed in our study. Besides, we found that patients with cardiac injury displayed higher proportion of pericardial effusion than those without cardiac injury. Moreover, decreased LV GLS_4CH_ and RV FWLS were also correlated with the presence of pericardial effusion, suggesting that the presence of pericardial effusion or pericarditis have a major influence on the biventricualr strain values.

In addition to myocardial injury, RV function was predisposed to impairment owing to increased RV afterload from ARDS, hypoxic pulmonary vasoconstriction, pulmonary microthrombi, and endothelial and microvascular injury (26). RV dilation and dysfunction may also affect the LV function and aggravate LV dysfunction by ventricular interdependence and paradoxical septum. The reductions in LV GLS_4CH_ and RV FWLS are important in COVID-19 patients, as owing to overlapping symptoms of dyspnea, the diagnosis of myocardial involvement may be challenging. These findings are also particularly significant to the majority of COVID-19 patients with a normal LVEF.

### The Utility of Biventricular Longitudinal Strain During the Follow-Up Study

At 3-month follow-up after discharge, significant improvements in biventricular longitudinal strain were identified in our study, indicating that depressed LV and RV performance may be reversible on disease recovery when the acute inflammatory response waned. Consistent with our results, Li et al. showed that impaired LV function appeared to be reversible at 30-day follow-up study in 46 patients with SARS (19). In another follow-up observation of 11 COVID-19 patients with LV dysfunction, Dr. Churchill and colleagues demonstrated resolution of LV abnormalities after a median of 14 days (27). However, LVEF and conventional RV function parameters did not show significant improvements with therapy in our study. These findings suggests biventricular longitudinal strain may be more sensitive to detect subtle myocardial improvement compared to other standard echocardiographic parameters. Our results demonstrate the superiority of biventricular longitudinal strain over conventional echocardiographic indices during the follow-up in patients with COVID-19.

### The Prognostic Value of Biventricular Longitudinal Strain in COVID-19 Patients

To the best of our knowledge, this may be the first study to investigate whether biventricular longitudinal strain were associated with fatal outcomes in COVID-19 patients. Indeed, in the present study, patients with diminished LV GLS_4CH_ and RV FWLS were at higher risk of death. Our findings reveal that biventricular longitudinal strain serve as novel imaging biomarkers that predicts higher mortality in patients with COVID-19. Consistent with these results, our study previously revealed that RV FWLS was an independent predictor of poor outcomes in COVID-19 patients (18). Similarly, Argulian et al. showed that RV dilation was predictive of in-hospital mortality in patients with COVID-19. (28) Another observation was reported by Szekely et al. (21), which demonstrated increased RV end diastolic area was significantly associated with mortality.

In addition, LV GLS has presented additional prognostic significance over LVEF in a range of cardiovascular disorders (6, 29). However, the prognostic implication of LV GLS_4CH_ in COVID-19 patients remained unknown. Our findings showed that LV GLS_4CH_ was predictive of higher mortality in COVID-19 patients, whereas LVEF was not. This is in contradistinction to a recent study in patients with COVID-19 in Israel, which reported that lower LVEF was associated with mortality (21). However, in the previous study (21), patients were older, and have higher rate of male, hypertension, diabetes mellitus, and obesity. The current data indicates that LV GLS_4CH_ and RV FWLS are not only more sensitive markers of subclinical myocardial impairment, but also powerful and independent predictors of higher mortality. Therefore, biventricular longitudinal strain could help risk stratification of COVID-19 patients.

### Clinical Implications

LVEF is a key determinant in clinical decision-making in various diseases. However, it is relatively indiscriminant within the normal range. Novel biventricular longitudinal strain may be of particular clinical significance in COVID-19 patients with relatively normal LVEF. Our data showed LV GLS_4CH_ and RV FWLS, rather than LVEF, were strong predictors of higher risk of mortality. Furthermore, biventricular longitudinal strain can provide highly useful and clinically relevant information during the follow-up in patients with COVID-19. The present study revealed the important clinical implication of biventricular longitudinal strain, as measurements of LV GLS_4CH_ and RV FWLS are fast and non-invasive methods that can be easily obtained from bedside echocardiography. More importantly, they can identify subclinical myocardial impairment, help detect in higher risk of COVID-19 patients and serially follow patients.

### Limitations

Our study has several limitations that should be mentioned. First, as 2D-STE depends on image quality, severe and critically ill patients with inadequate echocardiographic images might have been underrepresented. Furthermore, 2D-STE analysis was performed using Qlab software in our study, so the results in the present study may not be apply to other software algorithms because 2D-STE parameters are hampered by inter-vendor variability. Although our study exclude dilated cardiomyopathy and old myocardial infarction that may significant lead to impaired biventricular longitudinal strain, patients had hypertension or coronary artery diseases, who had underlying medical condition that could have affected strain values. In addition, our study used the LV GLS_4CH_ rather than the LV GLS to estimate LV myocardial longitudinal function during the epidemic of COVID-19 to allow rapid image acquisition and reduce contagion exposure duration to healthcare worker. Another limitation was that only a small proportion of COVID-19 patients had follow-up echocardiographic data, though improvement in biventricular longitudinal strain was noted. Finally, the study was a single-center study with a relatively limited sample size. Therefore, further large multi-center studies are needed to confirm the results in the present study.

## Conclusions

Our study demonstrates that LV GLS_4CH_ and RV FWLS are independently predicative of higher mortality, providing incremental prognostic implications over conventional echocardiographic parameters in patients with COVID-19. We also identify that biventricular longitudinal strain provide highly relevant information regarding the recovery of cardiac function when the acute inflammatory response subsided. Therefore, biventricular longitudinal strain are valuable non-invasive parameters in risk stratification and serial follow-up of patients with COVID-19.

## Data Availability Statement

The original contributions presented in the study are included in the article/Supplementary Material, further inquiries can be directed to the corresponding author/s.

## Ethics Statement

The studies involving human participants were reviewed and approved by Medical Ethics Committee of Union Hospital, Tongji Medical College, Huazhong University of Science and Technology. Written informed consent for participation was not required for this study in accordance with the national legislation and the institutional requirements.

## Author Contributions

All authors listed have made a substantial, direct and intellectual contribution to the work, and approved it for publication.

## Conflict of Interest

The authors declare that the research was conducted in the absence of any commercial or financial relationships that could be construed as a potential conflict of interest.

## References

[1] Shi S Qin M Shen B Cai Y Liu T Yang F Association of cardiac injury with mortality in hospitalized patients with COVID-19 in Wuhan, China. JAMA Cardiol. (2020) 5:802–10. 10.1001/jamacardio.2020.0950 32211816 PMC7097841

[2] Guo T Fan Y Chen M Wu X Zhang L He T. Cardiovascular implications of fatal outcomes of patients with coronavirus disease 2019 (COVID-19). JAMA Cardiol. (2020) 5:811–8. 10.1001/jamacardio.2020.1017 32219356 PMC7101506

[3] Potter E Marwick TH. Assessment of left ventricular function by echocardiography: the case for routinely adding global longitudinal strain to ejection fraction. JACC Cardiovasc Imaging. (2018) 11:260–74. 10.1016/j.jcmg.2017.11.017 29413646

[4] Kalam K Otahal P Marwick TH. Prognostic implications of global LV dysfunction: a systematic review and meta-analysis of global longitudinal strain and ejection fraction. Heart. (2014) 100:1673–80. 10.1136/heartjnl-2014-305538 24860005

[5] Xie M Li Y Cheng TO Wang X Dong N Nie X. The effect of right ventricular myocardial remodeling on ventricular function as assessed by two-dimensional speckle tracking echocardiography in patients with tetralogy of fallot: a single center experience from China. Int J Cardiol. (2015) 178:300–7. 10.1016/j.ijcard.2014.10.027 25453412

[6] Kim HM Cho GY Hwang IC Choi HM Park JB Yoon YE. Myocardial strain in prediction of outcomes after surgery for severe mitral regurgitation. JACC Cardiovasc Imaging. (2018) 11:1235–44. 10.1016/j.jcmg.2018.03.016 29778855

[7] Li Y Wang T Haines P Li M Wu W Liu M. Prognostic value of right ventricular two-dimensional and three-dimensional speckle-tracking strain in pulmonary arterial hypertension: superiority of longitudinal strain over circumferential and radial strain. J Am Soc Echocardiogr. (2020) 33:985–94. 10.1016/j.echo.2020.03.015 32532643

[8] Mast TP Taha K Cramer MJ Lumens J van der Heijden JF Bouma BJ. The prognostic value of right ventricular deformation imaging in early arrhythmogenic right ventricular cardiomyopathy. JACC Cardiovasc. Imaging. (2019) 12:446–55. 10.1016/j.jcmg.2018.01.012 29550307

[9] Lang RM Badano LP Mor-Avi V Afilalo J Armstrong A Ernande L. Recommendations for cardiac chamber quantification by echocardiography in adults: an update from the American society of echocardiography and the European association of cardiovascular imaging. Eur Heart J Cardiovasc Imaging. (2015) 28:1–39.e14. 10.1016/j.echo.2014.10.003 25712077

[10] Guideline for the Diagnosis and Treatment of (2019) Novel Coronavirus (2019-nCoV) Infected Pneumonia. Available online at: http://news.cyol.com/app/2020-02/05/content_18353703.htm (accessed March 4, 2020). 10.1186/s40779-020-0233-6 PMC7003341 32029004

[11] Chen N Zhou M Dong X Qu J Gong F Han Y. Epidemiological and clinical characteristics of 99 cases of 2019 novel coronavirus pneumonia in Wuhan, China: a descriptive study. Lancet. (2020) 395:507–13. 10.1016/S0140-6736(20)30211-7 32007143 PMC7135076

[12] Huang C Wang Y Li X Ren L Zhao J Hu Y. Clinical features of patients infected with 2019 novel coronavirus in Wuhan, China. Lancet. (2020) 395:497–506. 10.1016/S0140-6736(20)30183-5 31986264 PMC7159299

[13] Yang X Yu Y Xu J Shu H Xia J Liu H. Clinical course and outcomes of critically ill patients with SARSCoV-2 pneumonia in Wuhan, China: a single-centered, retrospective, observational study. Lancet Respir Med. (2020) 8:475–81. 10.1016/S2213-2600(20)30079-5 32105632 PMC7102538

[14] Wang D Hu B Hu C Zhu F Liu X Zhang J. Clinical characteristics of 138 hospitalized patients with 2019 novel coronavirus-infected pneumonia in Wuhan, China. JAMA. (2020) 323:1061–9. 10.1001/jama.2020.1585 32031570 PMC7042881

[15] Gackowski A Lipczyńska M Lipiec P Szymański P. Expert opinion of the working group on echocardiography of the polish cardiac society on performing echocardiographic examinations during COVID-19 pandemic. Kardiol Pol. (2020) 78:357–63. 10.33963/KP.15265 32241097

[16] Patel RB Vaduganathan M Greene SJ Butler J. Nomenclature in heart failure: a call for objective, reproducible, biologically-driven terminology. Eur J Heart Fail. (2018) 20:1379–81. 10.1002/ejhf.1231 29943879 PMC6179933

[17] Nauta JF Jin X Hummel YM Voors AA. Markers of left ventricular systolic dysfunction when left ventricular ejection fraction is normal. Eur J Heart Fail. (2018) 20:1636–8. 10.1002/ejhf.1326 30328663

[18] Li Y Li H Zhu S Xie Y Wang B He L. Prognostic value of right ventricular longitudinal strain in patients with COVID-19. JACC Cardiovasc Imaging. (2020) 13:2287–99. 10.1016/j.jcmg.2020.04.014 32654963 PMC7195441

[19] Li SS Cheng CW Fu CL Chan YH Lee MP Chan JW. Left ventricular performance in patients with severe acute respiratory syndrome. Circulation. (2003) 108:1798–803. 10.1161/01.CIR.0000094737.21775.32 14504188

[20] Løgstrup BB Nielsen JM Kim WY Poulsen SH. Myocardial oedema in acute myocarditis detected by echocardiographic 2D myocardial deformation analysis. Eur Heart J Cardiovasc Imaging. (2016) 17:1018–26. 10.1093/ehjci/jev302 26588987

[21] Szekely Y Lichter Y Taieb P Banai A Hochstadt A Merdler I. The spectrum of cardiac manifestations in coronavirus disease 2019 (COVID-19) - a systematic echocardiographic study. Circulation. (2020) 142:342–53. 10.1161/CIRCULATIONAHA.120.047971 32469253 PMC7382541

[22] Sud K Vogel B Bohra C Garg V Talebi S Lerakis S. Echocardiographic findings in COVID-19 patients with significant myocardial injury. J Am Soc Echocardiogr. (2020) 33:1054–5. 10.1016/j.echo.2020.05.030 32595004 PMC7274620

[23] Zhang L Wang B Zhou J Kirkpatrick J Xie M Johri AM. Bedside focused cardiac ultrasound in COVID-19 infection from the wuhan epicenter: the role of cardiac point of care ultrasound (POCUS), limited transthoracic echocardiography and critical care echocardiography. J Am Soc Echocardiogr. (2020) 33:676–82. 10.1016/j.echo.2020.04.004 32503706 PMC7144595

[24] Jain SS Liu Q Raikhelkar J Fried J Elias P Poterucha TJ. Indications and findings on transthoracic echocardiogram in COVID-19. J Am Soc Echocardiogr. (2020) 33:1278–84. 10.1016/j.echo.2020.06.009 32782131 PMC7298489

[25] Zheng YY Ma YT Zhang JY Xie X. COVID-19 and the cardiovascular system. Nat Rev Cardiol. (2020) 17:259–60. 10.1038/s41569-020-0360-5 32139904 PMC7095524

[26] Park JF Banerjee S Umar S. In the eye of the storm: the right ventricle in COVID-19. Pulm Circ. (2020) 10:2045894020936660. 10.1177/2045894020936660 32655856 PMC7333504

[27] Churchill TW Bertrand PB Bernard S Namasivayam M Churchill J Crousillat D. Echocardiographic features of COVID-19 illness and association with cardiac biomarkers. J Am Soc Echocardiogr. (2020) 33:1053–4. 10.1016/j.echo.2020.05.028 32580898 PMC7253994

[28] Argulian E Sud K Vogel B Bohra C Garg VP Talebi S. Right ventricular dilation in hospitalized patients with COVID-19 infection. JACC Cardiovasc Imaging. (2020) 13:2459–61. 10.1016/j.jcmg.2020.05.010 32426088 PMC7228729

[29] Caspar T Fichot M Ohana M El Ghannudi S Morel O Ohlmann P. Late detection of left ventricular dysfunction using two-dimensional and three-dimensional speckle-tracking echocardiography in patients with history of nonsevere acute myocarditis. J Am Soc Echocardiogr. (2017) 30:756–62. 10.1016/j.echo.2017.04.002 28599827

