# Supplementary material for: Biventricular Longitudinal Strain Predict Mortality in COVID-19 Patients
Source: Front Cardiovasc Med. 2021 Jan 18;7:632434. doi: 10.3389/fcvm.2020.632434 (PMC7848071; doi:10.3389/fcvm.2020.632434)
Supplement: Supplementary file 1 [file Data_Sheet_1.docx]

**
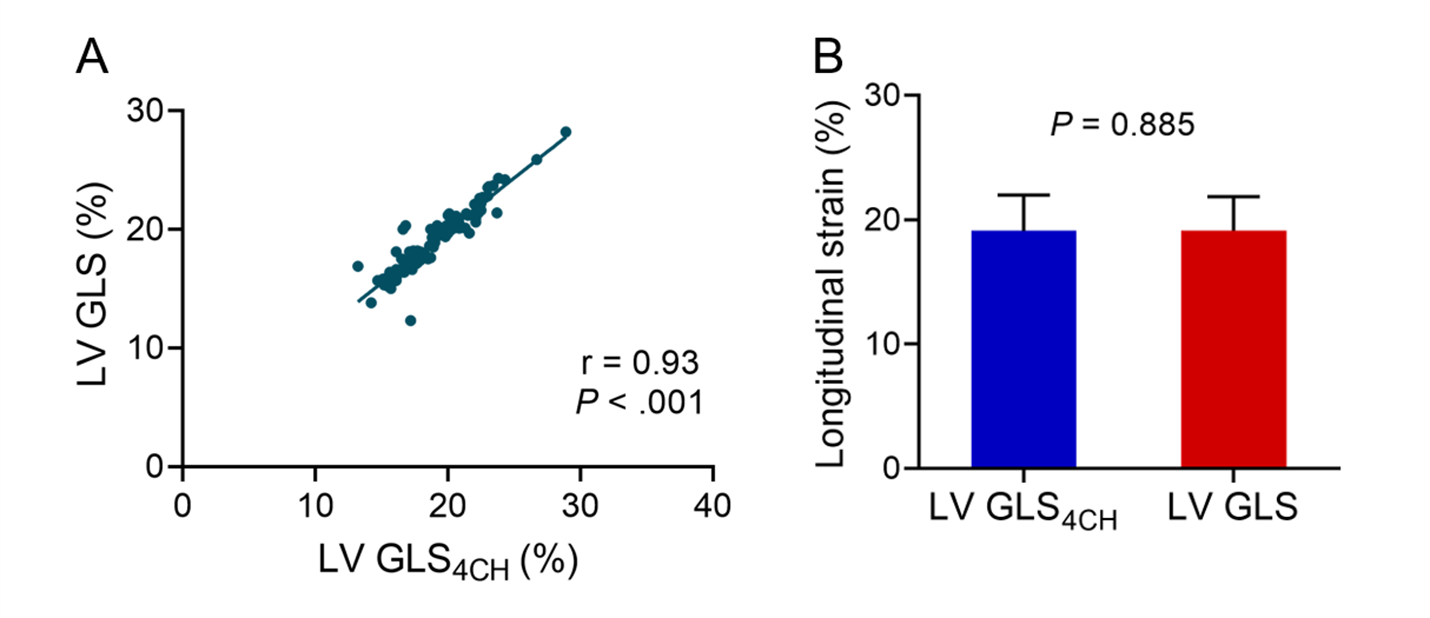
**

**Figure S1 Correlation or comparison between** **LV GLS_4CH_ and LV GLS.** LV GLS_4CH_ strongly correlated with LV GLS (A). There was no significant difference between LV GLS_4CH_ and LV GLS (B).

**
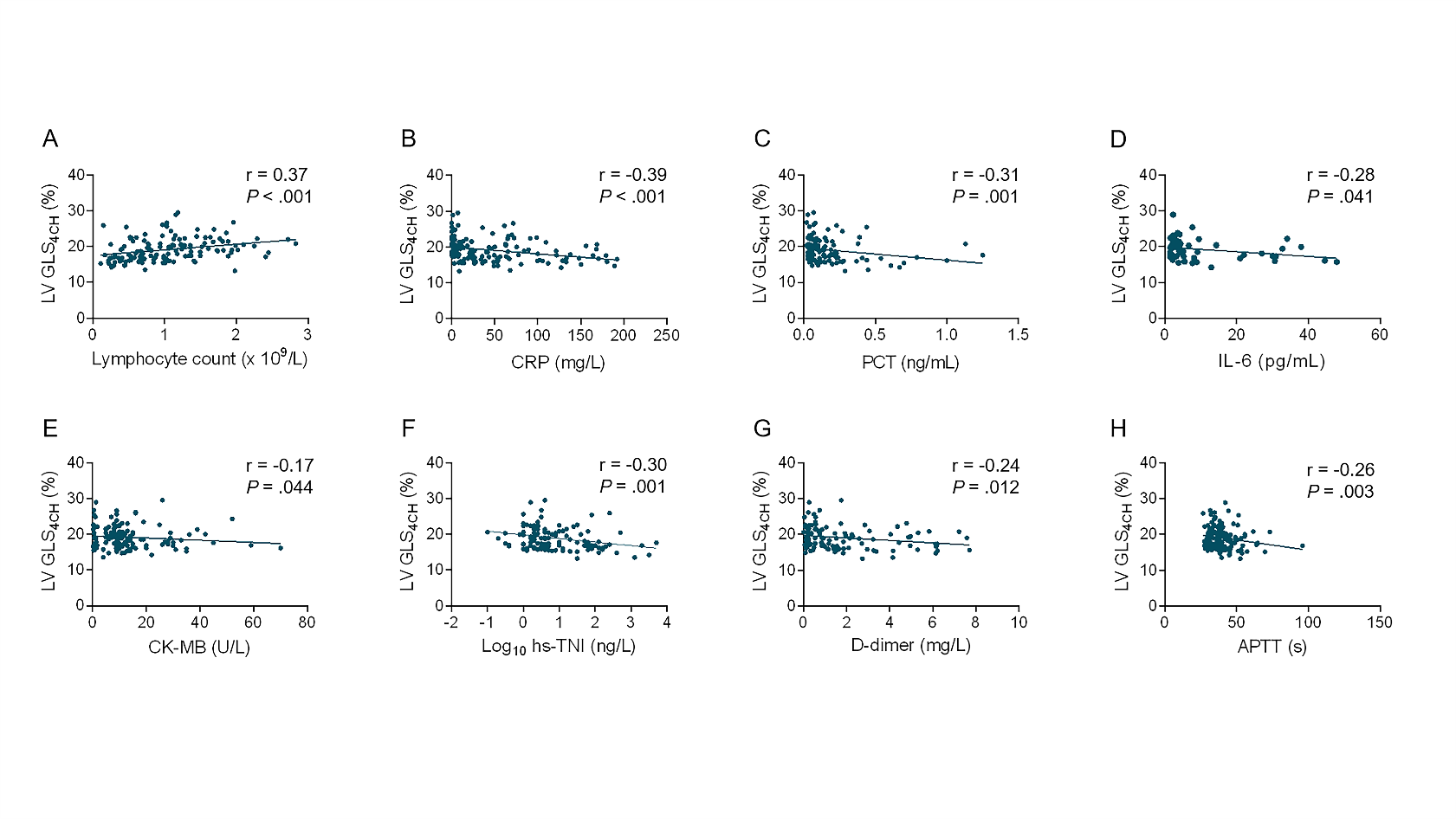
**

**Figure S2. Correlations of Left Ventricular Global Longitudinal Strain with Biomarkers Levels of Inflammation, Cardiac Injury and Coagulopathy.** Associations between left ventricular global longitudinal strain (LV GLS_4CH_) and lymphocyte count (A), C-reactive protein (CRP) (B), procalcitonin (PCT) (C), interleukin-6 (IL-6) (D), creatine kinase muscle-brain (CK-MB) (E), high-sensitivity troponin I (hs-TNI) (F), D-dimer (G), and activated partial thromboplastin time (APTT) (H). LV GLS_4CH_ values are absolute values.


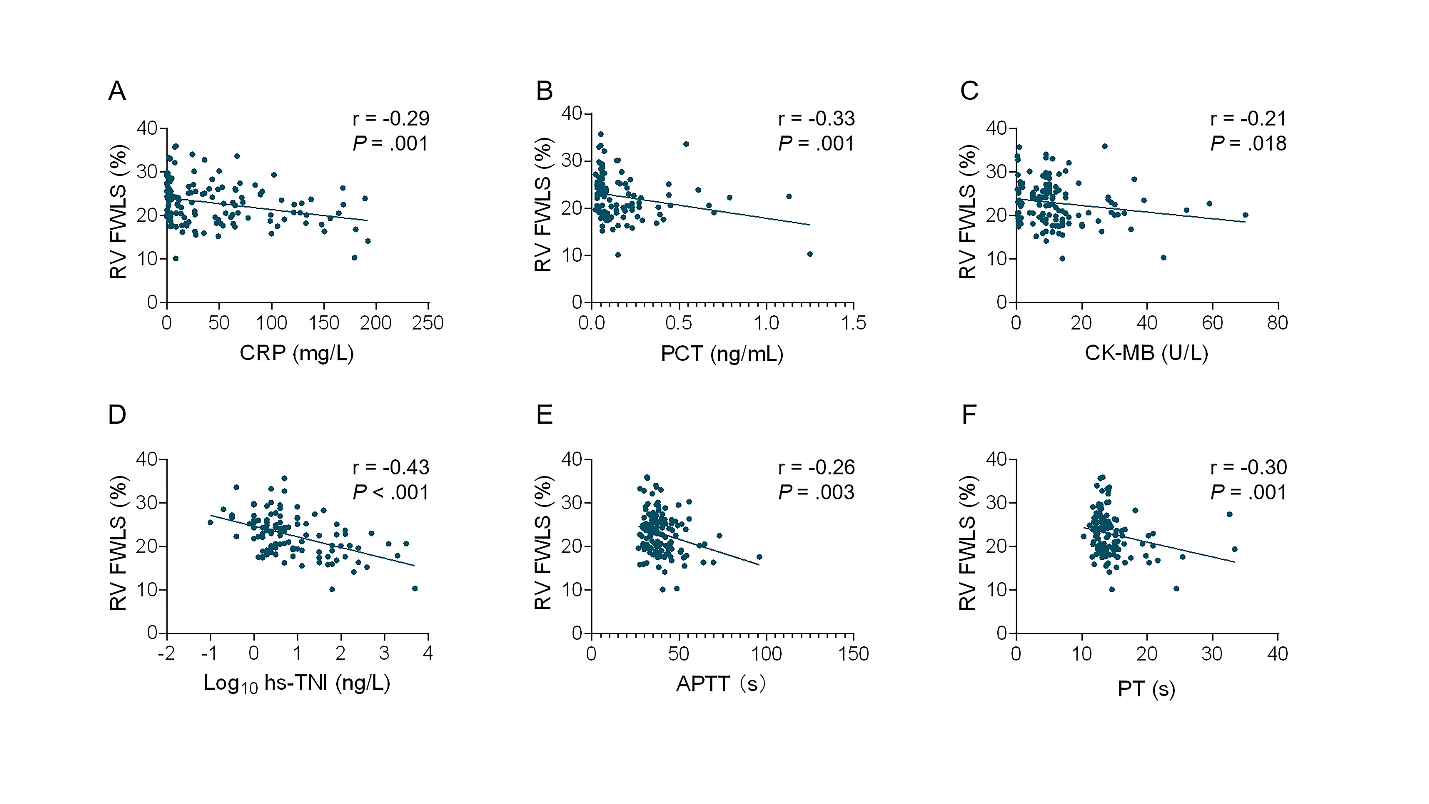


**Figure S3. Correlations of Right Ventricular Free Wall Longitudinal Strain with Biomarkers Levels of Inflammation, Cardiac Injury and Coagulopathy.** Associations between Right ventricular free wall longitudinal strain (RV FWLS) and C-reactive protein (CRP) (A) , procalcitonin (PCT) (B), creatine kinase muscle-brain (CK-MB) (C), high-sensitivity troponin I (hs-TNI) (D), activated partial thromboplastin time (APTT) (E), and prothrombin time (PT) (F). RV FWLS values are absolute values.

| **Table S1. Echocardiographic Characteristics of Patients With COVID-19 According to Mechanical Ventilation Therapy** | | | | | | | | | |
| --- | --- | --- | --- | --- | --- | --- | --- | --- | --- |
| **Variables** | | **All Patients**  **(n = 132)** | | **Without MV**  **(n=100)** | | **With MV**  **(n=32)** | | **p Value** | |
| **Left heart** | |  | |  | |  | |  | |
| LA dimension, mm | | 34.3±5.4 | | 34.2±5.3 | | 35.4±5.7 | | 0.280 | |
| LV dimension, mm | | 45.5±4.9 | | 45.4±4.9 | | 46.5±4.5 | | 0.215 | |
| IVS, mm | | 9.6±1.3 | | 9.7±1.1 | | 9.4±1.9 | | 0.242 | |
| PW, mm | | 8.8±1.9 | | 8.8±1.9 | | 9.1±1.9 | | 0.527 | |
| LVM, g | | 144.3±36.1 | | 144.8±36.3 | | 146.5±36.3 | | 0.818 | |
| EDT, ms | | 204.4±53.7 | | 206.7±55.4 | | 196.6±44.8 | | 0.353 | |
| E/A ratio | | 0.9±0.4 | | 0.9±0.4 | | 0.9±0.3 | | 0.442 | |
| E/e' ratio | | 9.1±3.2 | | 8.8±3.0 | | 9.9±3.5 | | 0.159 | |
| LVEDVI, ml/m^2^ | | 52.2±16.1 | | 52.0±15.0 | | 53.5±19.8 | | 0.743 | |
| LVESVI, ml/m^2^ | | 19.6±7.5 | | 19.5±7.1 | | 19.8±8.0 | | 0.920 | |
| LVEF, % | | 62.8±6.9 | | 63.0±7.1 | | 63.0±6.5 | | 0.945 | |
| LV GLS_4CH_, % | | 19.2±3.2 | | 19.7±3.1 | | 17.3±2.7 | | <0.001 | |
| LAS-peak, % | | 33.7±7.6 | | 33.2±7.4 | | 31.5±8.8 | | 0.336 | |
| Moderate-severe MR, n (%) | | 2(1.4%) | | 1(1.0%) | | 1(3.1%) | | 0.427 | |
| **Right heart** |  | |  | |  | |  | |  |
| RA dimension, mm | | 35.5±4.6 | | 35.4±4.7 | | 36.0±4.5 | | 0.595 | |
| RV dimension, mm | | 33.9±4.4 | | 33.7±4.3 | | 34.6±4.5 | | 0.365 | |
| TAPSE, mm | | 22.2±3.8 | | 22.5±3.8 | | 20.6±3.4 | | 0.015 | |
| RVFAC, % | | 46.9±6.6 | | 47.8±6.6 | | 44.4±5.7 | | 0.008 | |
| S’, cm/s | | 13.3(11.9,15.0) | | 13.6(12.0,15.0) | | 13.3(11.0,15.3) | | 0.735 | |
| RV FWLS, % | | 22.7(19.2,25.6) | | 23.6(20.1,26.3) | | 19.6(16.6,23.7) | | <0.001 | |
| Moderate-severe TR, n (%) | | 4(3.0%) | | 2(2.0%) | | 2(6.3%) | | 0.247 | |
| PASP, mm Hg | | 36±14 | | 31±10 | | 48±15 | | <0.001 | |
| Values are mean ± SD, n (%), median (interquartile range). COVID-19 = coronavirus disease 2019; EDT = peak E deceleration time of mitral inflow; IVS = interventricular septum; LA = left atrial; LAS = left atrial strain; LV = left ventricular; LV GLS_4CH_ = left ventricular global longitudinal strain derived from apical four-chamber view; LVEDVI = left ventricular end diastolic volume index; LVEF = left ventricular ejection fraction; LVESVI = left ventricular end systolic volume index; LVM = left ventricular mass; MR = mitral regurgitation; MV = mechanical ventilation; PASP = pulmonary artery systolic pressure; PW = posterior wall of left ventricular; RA = right atrial; RV = right ventricular; RV FWLS = right ventricular free wall longitudinal strain; RVFAC = RV fractional area change; TAPSE = tricuspid annular plane systolic excursion; TR = tricuspid regurgitation. LV GLS_4CH_ and RV FWLS values are absolute values. | | | | | | | | | |

| **Table S2. Intraobserver and interobserver reproducibility** | | | |
| --- | --- | --- | --- |
|  | ICC (95% CI) | Bias | Limits of agreement |
| Intraobserver |  |  |  |
| LV GLS_4CH_, % | 0.98(0.96,0.99) | 0.15 | -1.66～1.96 |
| LAS-peak, % | 0.93(0.84,0.97) | 0.26 | -3.73～4.24 |
| RV FWLS | 0.97(0.92,0.99) | 0.29 | -1.78～2.37 |
| Interobserver |  |  |  |
| LV GLS_4CH_, % | 0.94(0.93,0.99) | 0.27 | -2.47～3.00 |
| LAS-peak, % | 0.89(0.74,0.95) | 0.74 | -4.42～5.90 |
| RV FWLS | 0.88(0.71,0.95) | 0.31 | -2.87～3.49 |
| CI = confidence interval; ICC = intraclass correlation coefficient; LAS = left atrial strain; LV GLS_4CH_ = left ventricular global longitudinal strain derived from apical four-chamber view; RV FWLS = right ventricular free wall longitudinal strain. | | | |
